# Quantitative Evaluation of EEG-Biomarkers for Prediction of Sleep Stages

**DOI:** 10.3390/s22083079

**Published:** 2022-04-17

**Authors:** Iqram Hussain, Md Azam Hossain, Rafsan Jany, Md Abdul Bari, Musfik Uddin, Abu Raihan Mostafa Kamal, Yunseo Ku, Jik-Soo Kim

**Affiliations:** 1 Department of Biomedical Engineering, Medical Research Center, College of Medicine, Seoul National University, Seoul 03080, Korea; iqram20@snu.ac.kr; 2 Network and Data Analysis Group, Department of Computer Science and Engineering, Islamic University and Technology (IUT), Gazipur 1704, Bangladesh; azam@iut-dhaka.edu (M.A.H.); rafsanjany@iut-dhaka.edu (R.J.); abdulbari@iut-dhaka.edu (M.A.B.); musfikuddin@iut-dhaka.edu (M.U.); raihan.kamal@iut-dhaka.edu (A.R.M.K.); 3 Department of Biomedical Engineering, College of Medicine, Chungnam National University, Daejeon 35015, Korea; yunseo.ku@cnu.ac.kr; 4 Department of Computer Engineering, Myongji University, Yongin 17058, Korea

**Keywords:** electroencephalogram, sleep stages, physiological biomarker, neuroscience, polysomnography, machine-learning, sleep monitoring

## Abstract

Electroencephalography (EEG) is immediate and sensitive to neurological changes resulting from sleep stages and is considered a computing tool for understanding the association between neurological outcomes and sleep stages. EEG is expected to be an efficient approach for sleep stage prediction outside a highly equipped clinical setting compared with multimodal physiological signal-based polysomnography. This study aims to quantify the neurological EEG-biomarkers and predict five-class sleep stages using sleep EEG data. We investigated the three-channel EEG sleep recordings of 154 individuals (mean age of 53.8 ± 15.4 years) from the Haaglanden Medisch Centrum (HMC, The Hague, The Netherlands) open-access public dataset of PhysioNet. The power of fast-wave alpha, beta, and gamma rhythms decreases; and the power of slow-wave delta and theta oscillations gradually increases as sleep becomes deeper. Delta wave power ratios (DAR, DTR, and DTABR) may be considered biomarkers for their characteristics of attenuation in NREM sleep and subsequent increase in REM sleep. The overall accuracy of the C5.0, Neural Network, and CHAID machine-learning models are 91%, 89%, and 84%, respectively, for multi-class classification of the sleep stages. The EEG-based sleep stage prediction approach is expected to be utilized in a wearable sleep monitoring system.

## 1. Introduction

Sleep is a biological activity that occurs spontaneously in humans and has an influence on task performance, physical and mental health, and overall quality of life. Sleep accounts for about one-third of an individual’s whole lifetime. Sleep deprivation is the root cause of insomnia, anxiety, schizophrenia, and other mental illnesses. Moreover, drowsiness, an outcome of sleep deprivation, is a reason for around one-fifth of vehicle accidents and injuries. Sleep is a dynamic phenomenon including a variety of sleep phases, wake (W), non-rapid eye movement (NREM) sleep, and rapid eye movement (REM) sleep. Furthermore, NREM sleep stages are classified into NREM-1 (N1), NREM-2 (N2), and NREM-3 (N3) [1]. A healthy sleeper goes through multiple NREM and REM cycles throughout the night. The N1 stage occurs when the individual feels sleepy and marks the shift from the awake state. In Stage N2, the dynamics of vital signals, such as ocular movements, heart rate, body temperature, and brain activity start to attenuate. The N3 stage is considered deep sleep or slow-wave sleep; no eye or muscular movement occurs, and muscles and tissues are healed. The last stage, referred to as the REM state, is characterized by rapid eye movements and fast breathing. During this period, the body becomes relaxed, and dreaming begins. About 75% of the sleeping period is spent in NREM sleep, while REM sleep accounts for about 25% [2].

Physiological signal monitoring can be employed for health monitoring, disease prognostics, and functional response in daily activities, such as sleeping, driving, walking, working, and so on [3,4,5,6,7,8,9,10]. Tracking brainwaves is one of the essential methods for assessing cognitive load, and electroencephalography (EEG) is the physiological tool for measuring the electrical potential from the scalp that directly reflects the activities originating from the brain [11,12,13,14]. Polysomnography (PSG) has traditionally been used in the clinical environment to examine sleep quality and sleep disorders. PSG is a multi-sensor recording technique that collects physiological signals from the sleeping individual and is considered the primary tool in the diagnosis of sleep disorders. These biomedical signals include EEG, electromyography (EMG), electrooculography (EOG), and electrocardiography (ECG) [15]. These biosignals are measured to understand physiological activity, such as EEG for brain activity, EOG to track eye movement, EMG to measure the activity of muscles, and ECG to monitor the electrical activity of the heart [15]. Each 30-second epoch of the PSG signal is analyzed and given a sleep stage by a sleep specialist. The American Association of Sleep Medicine (AASM) classifies sleep-wake cycles into five phases: waking (W), REM sleep, and three types of NREM sleep: N1, N2, and N3. Moreover, the N1 and N2 stages are considered light sleep, and N3 is considered deep-sleep or slow-wave sleep [1].

The gold-standard PSG sleep scoring procedure is time-consuming and labor-intensive; it requires a human expert to score a whole night of sleep data manually by examining signal patterns. Additionally, a patient must attend a lab or clinic and spend the whole night PSG recording in a clinical environment, which is often an expensive and complex process. Aside from that, the PSG signal is very inconvenient and uncomfortable for individuals since its highly sticky electrodes and cabling are continually connected to the body. These challenges compelled physicians to depend only on subjective questionaries to determine sleep quality for neurological therapy and a variety of sleep disorder diagnostic methods. Therefore, developing an automated sleep staging system that is simple to use and reliable would have a significant contribution to this field [16,17,18,19]. HeathSOS, a wearable health monitoring system consisting of an eye mask embedded with EEG and EOG electrodes, has been reported as an alternative system for sleep monitoring [12,18]. Big-ECG, a cyber-physical cardiac monitoring system consisting of a wearable ECG sensor, a big data platform, and health advisory services, has been studied for disease prediction in the resting state, sleep, and other daily activities [15]. Several studies have reported that EEG signals are more helpful during sleep scoring than any other kind of PSG signal [20]. EEG signals directly track the brain’s activity and differentiate various sleep patterns [8,18,19].

Our study attempts to automate this sleep scoring process by utilizing data from three representative EEG channels from three cortical positions (F4, C4, and O2). F4 is representative of the frontal lobe, C4 is representative of the central lobe, and O2 is representative of the occipital lobe. We hypothesized that sleep-stage dependent responses of the central nervous system would be immediately sensed by the EEG. Signal processing, feature extraction, and a machine-learning approach are likely reliable methods to explore the physiological and neurological patterns of sleep stages.

We aim to investigate the EEG activity and identify the physiological biomarkers during sleeping. We developed the neurological state prediction model to classify the neurological responses in different phases of sleep. The key contributions of this paper can be summarized as follows:

- EEG biomarkers, consisting of frequency spectral measures for sleep stages, have been identified using statistical analysis.
- Machine-learning models have been developed to classify the neurological states in different sleep stages.

We organized the remainder of this article into four sections. The datasets and the methodology for EEG pre-processing, feature extraction, and statistical and machine-learning analysis methods are described in Section 2. After that, the results are reported in Section 3, trailed by the discussion. Lastly, we state our conclusions in Section 5.

## 2. Materials and Methods

**Figure 1:**
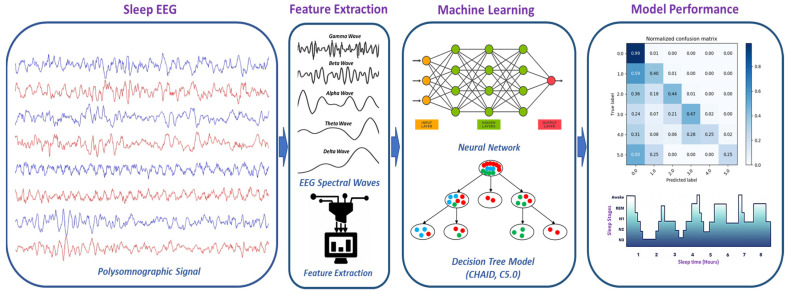
Methodology of EEG-based sleep stages classification using a machine-learning approach.

To identify the physiological biomarkers of sleep stages and develop a machine-learning-based prediction model to classify the sleep stages, we performed EEG data pre-processing, feature extraction, feature selection, statistical analysis of features, and a machine-learning classification approach (Figure 1). Details about the EEG data processing, statistical analysis, and machine-learning classification methods are presented in the following subsections.

### 2.1. Dataset

We utilized the sleep recording of Haaglanden Medisch Centrum (HMC, The Hague, The Netherlands), available as an open-access public dataset in PhysioNet [21,22]. It was collected in 2018 and published very recently on 1 July 2021. The dataset includes a whole-night PSG sleep recording of 154 people (88 Male, 66 Female) with a mean age of 53.8 ± 15.4 years. Patient recordings were chosen at random and represented a diverse group of people who were referred for PSG examinations in the context of various sleep disorders. All signals were captured at 256 Hz using AgAgCl electrodes on SOMNOscreen PSG, PSG+, and EEG 10–20 recorders (SOMNOmedics, Randersacker, Germany). Each recording consists of four-channel EEG (F4/M1, C4/M1, O2/M1, and C3/M2), two-channel EOG (E1/M2 and E2/M2), one-channel bipolar chin EMG, and one-channel ECG. The recordings also contain the sleep scoring, consisting of W, N1, N2, N3, and R for an epoch of 30 sec. The AASM guidelines were used to score sleep stages which were manually scored by well-trained sleep technicians [1]. We have decided to use three EEG channels (F4, C4, and O2) in this study according to the international 10–20 EEG system.

### 2.2. Pre-Processing

The EEG signal was filtered to remove any 60 Hz AC noise from the nearby electrical grid. The eye-blink and muscle artifacts were separated and removed using EOG and EMG recordings from the EEG signal. Independent component analysis (ICA) was then used to eliminate ocular and muscle artifacts from the EEG signal using the FastICA methods [23]. Low-frequency motion artifact interference was produced by head and sensor movement close to the skin. A signal-to-noise ratio (SNR) was obtained for each signal by calculating the power ratio of the movement-affected EEG signal and the undisturbed measurement [24]. A band-pass filter was used to filter the EEG waveform within the frequency range of 0.5–44 Hz. The pre-processing and feature extraction of EEG data were carried out using the AcqKnowledge version 5.0 program (Biopac Systems Inc., Goleta, CA, USA).

### 2.3. Feature Extraction

EEG can be defined in terms of frequency and power within different frequency bands. The delta (δ) band ranges in frequency from 0.5 to 4.0 Hz, the theta (θ) band ranges in frequency from 4.0 to 8.0 Hz, the alpha (α) wave runs on 8.0–13.0 Hz, the beta (β) band is maintained in frequency from 13.0 to 30.0 Hz, and the gamma (γ) wave attained 30.0–44.0 Hz band [25,26]. EEG features were extracted from EEG signals using Fast Fourier transforms (FFT) and other methods to study the power within the EEG data. The power spectrum was computed using power spectral density (PSD) for each time epoch using the Welch periodogram technique [27]. For each epoch, the mean power, median frequency, mean frequency, spectral edge, and peak frequency features were extracted from this PSD. The epoch width was specified as 30 s. Extracted EEG features are summarized in Table 1 of this study. This EEG dataset contains a total of 89 sets of EEG features.

#### 2.3.1. EEG Frequency-Domain Features

The EEG Frequency Analysis was performed using FFT and the Welch periodogram [27] on artifact-free EEG signals with 10% hamming and extracted absolute power in the following spectral frequency bands: delta (0.5–4.0 Hz), theta (4.0–8.0 Hz), alpha (8.0–13.0 Hz), beta (13.0–30 Hz), and gamma (30.0–44 Hz). The average power of the power spectrum within the epoch was defined as the mean power. The median frequency was defined as the frequency at which half of the total power in the epoch is attained. The mean frequency was defined as the frequency at which the epoch’s average power is obtained. The spectral edge is defined as the frequency below which 90% of the total power inside the epoch is attained. The frequency at which the maximum power occurs throughout the epoch was identified as the peak frequency. To normalize the amplitudes of distinct EEG bands, relative power (RP) was computed as the ratio of each band’s power to the total power of all bands. For every 30 s epoch, all band power features were calculated. The following is the definition of the spectral power density of an EEG time-series signal x(t) with frequency j:

(1) $$E_{j}=\lim_{t\to \infty }\frac{1}{t}{\left|\hat{x_{t}}\left(j\right)\right|}^{2}$$

where $\hat{x_{t}}\left(j\right)$ is the Fourier transform of x(t) at frequency, j (in Hz) using the Welch periodogram. The EEG Band Relative power is defined in Equation (2).

(2) $$e_{j}=\frac{E_{\left(j_{1},j_{2}\right)}}{\sum_{j=0.5}^{44}E_{j}}$$

where E_j_ is absolute spectral power density with frequency j (with j = 0.5,..., 44) and j_1_ and j_2_ are the low and high frequency in Hz, respectively, and (j_1_, j_2_) is defined as δ (0.5, 4), θ (4, 8), α (8, 13), β (13, 30), and γ (30.0, 44) [28].

#### 2.3.2. DAR, DTR, and DTABR

The delta-alpha ratio (DAR), defined as the ratio of delta to alpha band power, was calculated according to Equation (3). The delta-theta ratio (DTR) was defined as the ratio of delta band power to theta band power and computed according to Equation (4). Equation (5) defines the (Delta + Theta)/(Alpha + Beta) ratio (DTABR), identified as the relative sum of slow-wave (delta rhythm and theta rhythm) power to fast-oscillating wave (alpha rhythm and beta rhythm) power [29].

(3) $$\mathrm{DAR}=\frac{e_{j=\delta }}{e_{j=\alpha }}$$

(4) $$\mathrm{DTR}=\frac{e_{j=\delta }}{e_{j=\theta }}$$

(5) $$\mathrm{DTABR}=\frac{e_{j=\delta }+e_{j=\theta }}{e_{j=\alpha }+e_{j=\beta }}$$

where j is the spectral frequency range, delta (δ) ranges 0.5–4.0 Hz, theta (θ) ranges 4.0–8.0 Hz, and alpha (α) ranges 8.0–13.0 Hz; $e_{j=\delta }$ and $e_{j=\alpha }\;$is the EEG Band Relative delta and alpha power, respectively, in different sleep stages (W, N1, N2, N3, and R).

### 2.4. Features Selection

Feature selection greatly reduces the time and memory required for data processing, enabling machine learning algorithms to focus on just the most important features. The F-statistics [30] were used to determine the relevance of each feature on a scale ranging from zero to one. We used the *p*-value (probability) based on F-statistics for feature selection to investigate the most contributing features after performing the one-way ANOVA F-test for each continuous predictor. In the first step, we eliminated any features that had constant or missing values. The significance of each feature was measured by its effectiveness in independently predicting the target class. In this study, features with feature importance (1-p) of more than 95% were selected, where *p* is the F-test result.

### 2.5. Classification Algorithms

Machine-learning algorithms were used to classify neurological features during wakefulness, stages N1, N2, N3, and R. EEG feature data from 80% of selected features was used for training, while 20% of data were used for testing classification algorithms. The Neural Network, CHAID, and C5.0 models were used to distinguish the neurological features of sleep stages. As the N1 stage dataset is smaller compared with the other sleep stage datasets, we implemented the “class weighting” technique [31], heavily weighting the N1 stage and under-weighting the majority classes to deal with the imbalanced dataset.

#### 2.5.1. The Neural Network Model

The neural network is a data analysis technique that makes predictions based on the growth of a complex multi-layered network [32]. In this research, we employed a multilayer perceptron (MLP) neural network. This model is capable of estimating a broad variety of analytical models with minimal requirements on the model structure and assumptions. This model is comprised of multiple input nodes, a neural network with hidden layers, and an output layer.

#### 2.5.2. Chi-Squared Automatic Interaction Detector (CHAID) Model

The chi-squared automatic interaction detector (CHAID) method creates a decision tree by incrementally breaking a subset into two or more child nodes, starting with the entire data set [33]. The optimal partition across all nodes is obtained by merging the classifiers’ pairs until no significant difference in the target’s pair is noticed. As a decision tree model, the output of the CHAID model is visually appealing and simple to read in a clinical decision support system. This technique is commonly used in applications involving biological data analysis.

#### 2.5.3. C5.0 Model

The C5.0 model is a supervised data analysis method that attempts to construct decision trees or rule sets [34]. This model partitions the data according to the field with the greatest gain ratio. The model constructs a decision tree which is then pruned to reduce the tree’s estimated error rate. This model requires little training time and is resilient to missing data and a large number of input variables.

### 2.6. Data Analysis

This study employed descriptive statistics to compare the participants’ demographic data. The characteristics of the EEG spectra features were shown in a bar chart with an error bar. The data in the bar chart represents the mean value of the data along with their respective 95% confidence intervals (CI). Methods of statistical analysis consisted of descriptive statistics and hypothesis tests. The independent-samples *t*-test was used as a comparative measure of EEG features among sleep stages. A *p*-value of less than 0.05 was marked as statistically significant. Statistical analyses were accomplished using SPSS 26 software (IBM, Armonk, NY, USA). For the classification of sleep phases, we utilized state-of-art machine learning methods. EEG feature datasets were partitioned into the training and the testing dataset. We trained the machine learning algorithms on the training dataset to build the classification models which were then utilized for prediction on the EEG testing datasets. To eliminate overfitting, we used non-exhaustive k-fold (k = 10) cross-validation on the training dataset. For machine learning evaluations, we utilized IBM SPSS Modeler 18 software (IBM, Armonk, New York, NY, USA).

## 3. Results

### 3.1. Statistical Analysis

#### 3.1.1. EEG Biomarkers for Sleep Stages

**Figure 2:**
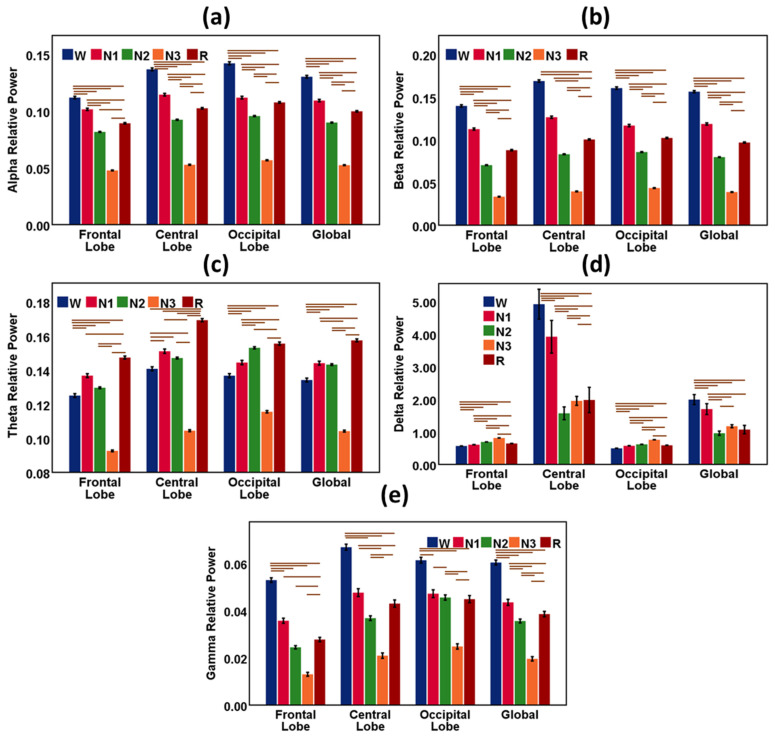
Results from EEG spectral power features during sleep stages W, N1, N2, N-3, and R. The bar chart describes the relative mean power of the EEG waves, and the vertical error bar (black color) is the 95% CI. (**a**) Alpha relative power for sleep stages in the frontal lobe, central lobe, occipital lobe, and global. (**b**) Beta relative power for sleep stages in the frontal lobe, central lobe, occipital lobe, and global. (**c**) Theta relative power for sleep stages in the frontal lobe, central lobe, occipital lobe, and global. (**d**) Delta relative power for sleep stages in the frontal lobe, central lobe, occipital lobe, and global. (**e**) Gamma relative power for sleep stages in the frontal lobe, central lobe, occipital lobe, and global. Global indicates the average measures of features of the frontal, central, and occipital lobes. The horizontal bars (brown color) are the outcomes of the hypothesis tests and indicate significant differences (*p* < 0.05) in EEG features among the sleep stages.

The EEG waveform varied during sleep with the change in sleep stages. Figure 2 shows the bar charts with error bars with a 95% confidence interval (C.I.) of EEG features of frequency bands during sleep stages W, N1, N2, N-3, and R. The global data indicates the average measures of the features of the frontal, central, and occipital lobes. The horizontal bars (brown color) are the outcomes of the hypothesis tests and indicate significant differences (*p* < 0.05) in EEG features among the sleep stages. 

Alpha was highest in the wake stage and lowest in the N3 or deep sleep stage in all cortical positions. Alpha gradually weakens as sleep becomes deeper. In the REM sleep stage, the alpha wave again gains strength. Beta was also dominant in the wake stage and lowest in the N3 or deep sleep stage in all cortical positions. Beta gradually becomes dormant as sleep propagates from light sleep to a deep sleep state. In the REM sleep stage, the beta wave again increases.

Theta was highest in the REM stage and lowest in the N3 or deep sleep stage in all cortical positions. Theta increases in light sleep. In the REM sleep stage, the theta wave again gains strength. Delta was highest in deep sleep or N3 stage and lowest in the wake stage in the frontal and occipital cortical positions. An exception is observed only in the central lobe. In the central lobe, delta was highest in the wake stage and sharply went down in the N1 and N2 stages. Delta again gradually increased as sleep became deeper and was highest in REM sleep in the central cortex. Gamma was highest in the wake stage and lowest in the N3 or deep sleep stage in all cortical positions. Gamma gradually weakens as sleep becomes deeper. In the REM sleep stage, the gamma wave again became dominant. Statistical results (Mean and Standard Deviation) of EEG spectral features (δ, θ, α, β, and γ) during sleep stages are reported in Table 2.

#### 3.1.2. Association of DAR, DTR, and DTABR with Sleep Stages

**Figure 3:**
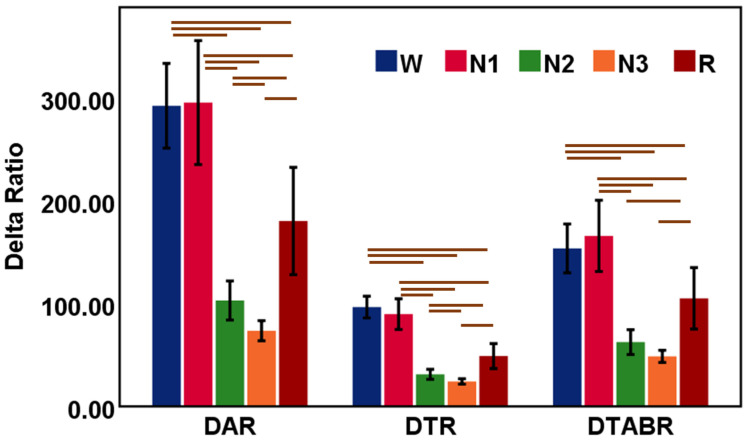
Results from DAR, DTR, and DTABR during sleep stages W, N1, N2, N-3, and R. The bar chart describes the relative mean power of the EEG waves and the vertical error bar (black color) is the 95% CI. Global indicates the average measures of features of the frontal, central, and occipital lobes. The horizontal bars (brown color) are the outcomes of the hypothesis tests and indicate significant differences (*p* < 0.05) in EEG features among the sleep stages.

Delta power ratios, such as DAR and DTR, were explored during sleep stages W, N1, N2, N-3, and R (Table 3). Figure 3 shows the bar charts with error bars with a 95% confidence interval of DAR, DTR, and DTABR in sleep stages. Global delta ratio parameters (DAR, DTR, and DTABR) were dominant in the wake and N1 stages; they decreased sharply in the N2 and N3 stages. In the REM sleep stage, DAR, DTR, and DTABR further increase compared with the deep sleep N3 stage.

### 3.2. Machine Learning Analysis

Machine-learning algorithms were utilized to predict the physiological states of various sleep stages. Machine Learning analysis is comprised of three steps: feature selection, model training, and model testing (or validation). During the feature selection process, the F-statistics were used to assess the feature relevance of sleep EEG features. EEG features with a *p*-value larger than 0.95 were selected for further classification investigation. The confusion matrix, also known as the error matrix, clearly demonstrates prediction outcomes for all target classes. Other performance parameters are computed using the confusion matrix, including accuracy, sensitivity, and precision. Accuracy was defined as the ratio of correct predictions to total observations and was regarded as the most intuitive performance metric for identifying the optimal model. The following standard formulas are used to estimate the performance evaluation matrix:

$$\mathrm{Sensitivity}=\frac{\mathrm{TP}}{\mathrm{TP}+\mathrm{FN}}$$

$$\mathrm{Specificity}=\frac{\mathrm{TN}}{\mathrm{TN}+\mathrm{FP}}$$

$$\mathrm{Precision}=\frac{\mathrm{TP}}{\mathrm{TP}+\mathrm{FP}}$$

$$\mathrm{Negative}\;\mathrm{predictive}\;\mathrm{value}\;\left(\mathrm{NPV}\right)=\frac{\mathrm{TN}}{\mathrm{TN}+\mathrm{FN}}$$

$$\mathrm{Accuracy}=\frac{\mathrm{TN}+\mathrm{TP}}{\mathrm{TN}+\mathrm{TP}+\mathrm{FN}+\mathrm{FP}}$$

where TP stands for the true positive, TN means the true negative, FP stands for the false positive, and FN means the false negative.

#### Multi-Class Classification of Sleep Stages

**Figure 4:**
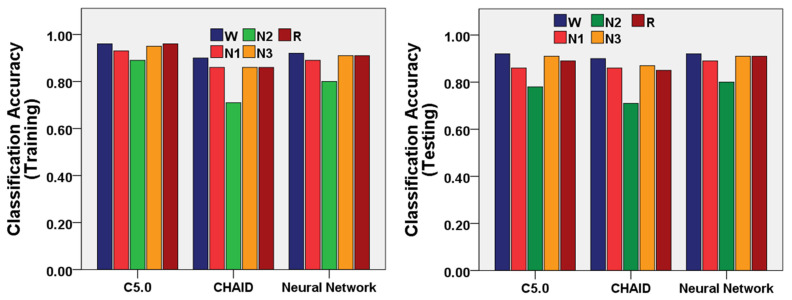
Performance of the three machine-learning models (C5.0, Neural Network, and CHAID Models) to classify the sleep stages W, N1, N2, N-3, and R using training and testing datasets of EEG features.

We utilized the machine learning algorithms for the multi-class classification of the sleep stages W, N1, N2, N-3, and R. The confusion matrices of the three machine-learning models (C5.0, Neural Network, and CHAID Models) were demonstrated in Table 4, Table 5 and Table 6 as the outcomes of prediction performance for sleep stages. The performances of the three machine-learning models (C5.0, Neural Network, and CHAID Models) were demonstrated in Figure 4 to classify the sleep stages using a training and testing dataset of EEG features.

The C5.0 model showed 94% accuracy using the training dataset and 87% accuracy using the testing dataset for multi-class classification of sleep stages (Table 7). N3 and W stages were most accurately classified with accuracy for training (95% and 96%) and testing (91% and 92%). The wake stage was classified with the highest sensitivity for training (92%) and testing (81%). The sensitivity of the C5.0 model was the lowest in the N1 stage. Moreover, the wake stage was classified with the highest precision for training (86%) and testing (77%). Furthermore, the negative predictive value of the C5.0 model was highest in the wake stage for training (98%) and testing (96%).

The Neural Network model showed 89% accuracy using the training dataset and 89% accuracy using the testing dataset for multi-class classification of sleep stages (Table 8). The N3, REM, and W stage were the most accurately classified with accuracy for training (91%, 91%, and 92%) and testing (91%, 91%, and 92%). The wake stage was classified with the highest sensitivity for training (86%) and testing (86%). The sensitivity of the Neural Network model was lowest for the N1 stage. Moreover, the wake stage was classified with the highest precision for training (75%) and testing (76%). Furthermore, the negative predictive value of the Neural Network model was highest in the wake stage for training (97%) and testing (97%).

The CHAID model showed 84% accuracy using the training dataset and 84% accuracy using the testing dataset for multi-class classification of sleep stages (Table 9). The W stage was most accurately classified with accuracy for training (90%) and testing (90%). The wake stage was classified with the highest sensitivity for training (72%) and testing (71%). The sensitivity of the CHAID model was lowest in the N1 stage. Moreover, the wake stage was classified with the highest precision for training (73%) and testing (73%). Furthermore, the negative predictive value of the CHAID model was highest in the wake stage for training (94%) and testing (94%).

## 4. Discussion

In our study, we characterized the neurological changes in sleep stages and classification of sleep stages using three EEG channels located in the frontal (F4), central (C4), and occipital (O2) lobes of a diverse group of adults. The extent of neurological change depends on the individual’s sleep pattern, dynamics of sleep stage transitions, and the individual’s lifestyle overall. We evaluated the neurological biomarkers through EEG in every sleep stage. Patient recordings were randomly chosen and reflected a broad sample of individuals referred for PSG exams for a variety of sleep disorders. Sleep is classified as REM or NREM sleep. Stages N1, N2, and N3 correspond to NREM sleep. Different sleep phases must be characterized and classified to identify sleep-related diseases. For instance, detecting REM sleep is an essential job for diagnosing REM sleep behavior disorder, and classification of wake and sleep states is required for sleep monitoring. This study addresses these demands by classifying W, N1, N2, N3, and REM stages.

Alpha rhythm, one of the basic features of human EEG, is prominent in the relaxed eye-closed awake state, N1, and REM sleep [35]. Alpha attenuates during high arousal states. In our study, alpha oscillation is higher in the resting awake state and decreases in the light sleep stage. The alpha activity also increases during REM sleep due to the short bursts of alpha rhythm [36]. A similar nature was observed for beta activity in sleep stages. Theta rhythm increases in light sleep (N1 and N2) stages relative to the wake stage and attenuates in the slow-wave sleep (N3) stage. A rise in delta activity was observed in the slow-wave deep-sleep (N3) stage compared with light sleep stages. Delta wave is considered an indicator of slow-wave deep sleep [37].

It has been observed that the classification rates for the N1 and N2 sleep stages are lower, which is one of the most challenging tasks. The N2 sleep stage is usually the transition between the light sleep and deep sleep stages [38]. As both N1 and N2 stages are light sleep states, the N2 stage is often mislabeled as N1. Therefore, the automated sleep staging algorithm misclassified it as N1 or N2 [39]. Moreover, gamma rhythms are identical in light sleep stages (N1 and N2) and REM sleep. This may also lead to the misclassification of N1, N2, and REM sleep stages. Furthermore, human sleep is a combination of distinct sleep phases with an unequal distribution of sleep epochs. Table 10 demonstrates a comparative study of methodologies and results between the current work and previous machine learning-based sleep studies. It is observed in Table 10 that our proposed approach has a notable improvement in prediction performance compared with the existing state-of-the-art works related to the five-class sleep states classification. Our classification performance is much higher than other multi-class classification studies.

We analyzed only three-channel EEG data to understand the neurological changes in EEG due to sleep stages, focusing on single-channel data from each frontal, central, and occipital lobe. Although a standard sleep study consists of multimodal biosignals, we did not study all EEG channels to simplify the automatic sleep stage prediction suitable for a wearable sleep monitoring system. In the future, we plan to extend this study with multimodal signals to enhance the accuracy of the prediction models.

## 5. Conclusions

Prediction of sleep stages is considered an assistive technology in machine-learning-enabled wearable sleep monitoring systems. The neurological biomarkers of sleep stages have been quantified through the EEG signal of polysomnography. In NREM sleep, attenuation of the alpha, beta, and gamma rhythms were observed, as well as the rise of theta and delta rhythms with the awake state and the subsequent increase in alpha and beta rhythms in REM sleep. Delta wave power ratios (DAR, DTR, and DTABR) are expected to be considered as biomarkers for their nature of decreasing NREM sleep and subsequent increase in REM sleep. The overall accuracy of the C5.0, Neural Network, and CHAID models are 91%, 89%, and 84%, respectively, in the multi-class classification of the sleep stages. This EEG-based sleep stage prediction technique is a promising candidate for further neuroscience research in a wearable sleep monitoring system.

## Figures and Tables

**Table 1:** Features extracted from the EEG signal. The Global channel is averaged over F4, C4, and O2 electrodes.

EEG Channel	EEG Spectral Waves	EEG Feature	Number of Features
F4, C4, and O2	δ, θ, α, β, and γ	Mean Power	15
F4, C4, and O2	δ, θ, α, β, and γ	Median Frequency	15
F4, C4, and O2	δ, θ, α, β, and γ	Mean Frequency	15
F4, C4, and O2	δ, θ, α, β, and γ	Spectral Edge	15
F4, C4, and O2	δ, θ, α, β, and γ	Peak Frequency	15
Global	δ, θ, α, β, and γ	Mean Power	5
F4, C4, and O2	DAR (δ/α) and DTR (δ/θ)	Mean Power	6
F4, C4, and O2	-	Total Mean Power	3

**Table 2:** Statistical results (Mean and Standard Deviation) of EEG spectral features (δ, θ, α, β, and γ) in the frontal, central, and occipital lobes during sleep stages W, N1, N2, N-3, and R. Global indicates the average measures of features of the frontal, central, and occipital lobes.

	EEG Feature	N1		N2		N3		R		W	
		Mean	Std. Dev.	Mean	Std. Dev.	Mean	Std. Dev.	Mean	Std. Dev.	Mean	Std. Dev.
**Frontal Lobe**	**Alpha**	0.102	0.056	0.082	0.042	0.048	0.028	0.089	0.042	0.112	0.079
	**Beta**	0.113	0.070	0.070	0.045	0.033	0.028	0.088	0.051	0.140	0.092
	**Theta**	0.137	0.064	0.130	0.051	0.093	0.037	0.147	0.058	0.125	0.078
	**Delta**	0.613	0.186	0.694	0.144	0.813	0.108	0.648	0.148	0.570	0.234
	**Gamma**	0.036	0.061	0.024	0.070	0.013	0.061	0.028	0.060	0.053	0.071
**Central Lobe**	**Alpha**	0.115	0.062	0.092	0.045	0.053	0.032	0.102	0.044	0.137	0.087
	**Beta**	0.126	0.075	0.083	0.048	0.040	0.033	0.100	0.050	0.169	0.097
	**Theta**	0.151	0.069	0.147	0.053	0.104	0.043	0.169	0.060	0.141	0.084
	**Delta**	3.922	27.494	1.572	19.725	1.954	10.219	1.982	25.349	4.922	32.353
	**Gamma**	0.048	0.092	0.037	0.101	0.021	0.085	0.043	0.101	0.067	0.092
**Occipital Lobe**	**Alpha**	0.112	0.064	0.096	0.046	0.057	0.032	0.108	0.048	0.142	0.097
	**Beta**	0.117	0.074	0.086	0.050	0.043	0.033	0.102	0.048	0.161	0.101
	**Theta**	0.144	0.071	0.153	0.064	0.116	0.052	0.156	0.060	0.137	0.086
	**Delta**	0.580	0.207	0.620	0.170	0.759	0.136	0.590	0.156	0.499	0.261
	**Gamma**	0.047	0.091	0.046	0.112	0.025	0.084	0.045	0.100	0.061	0.089
**Global**	**Alpha**	0.109	0.058	0.090	0.041	0.052	0.028	0.100	0.041	0.130	0.084
	**Beta**	0.119	0.070	0.080	0.045	0.039	0.029	0.097	0.046	0.156	0.090
	**Theta**	0.144	0.064	0.143	0.050	0.104	0.040	0.157	0.054	0.134	0.079
	**Delta**	1.701	9.200	0.960	6.591	1.175	3.422	1.071	8.468	1.994	10.825
	**Gamma**	0.043	0.071	0.036	0.084	0.020	0.068	0.038	0.076	0.060	0.075

**Table 3:** Statistical results (Mean and Standard Deviation) of EEG Delta features (DAR, DTR, and DTABR) in the Global cortex during sleep stages W, N1, N2, N-3, and R. Global indicates the average measures of features of the frontal, central, and occipital lobes.

	EEG Feature	N1		N2		N3		R		W	
		Mean	Std. Dev.	Mean	Std. Dev.	Mean	Std. Dev.	Mean	Std. Dev.	Mean	Std. Dev.
**Global**	**DAR**	296.0	3326.7	103.0	1917.7	73.6	723.5	180.5	3406.5	292.8	2914.9
	**DTR**	89.8	824.5	31.2	486.4	24.3	195.8	48.9	790.4	96.6	748.6
	**DTABR**	166.0	1912.7	62.5	1219.8	48.6	440.1	105.1	1950.1	153.8	1678.6

**Table 4:** Confusion matrix of the C5.0 Model using training and testing datasets for the classification of EEG features of the sleep stages W, N1, N2, N-3, and R.

C5.0		Prediction									
		N1	N2	N3	REM	Wake	N1	N2	N3	REM	Wake
**Actual**	**N1**	5760	1529	78	705	1451	748	632	42	383	585
	**N2**	837	27,581	1849	842	481	493	5713	858	548	226
	**N3**	88	2262	14,656	66	63	38	976	3083	38	20
	**REM**	665	1110	103	11,046	233	400	676	55	2060	117
	**Wake**	622	443	37	152	14,192	391	237	25	86	3170

**Table 5:** Confusion matrix of the Neural Network Model using training and testing datasets for the classification of EEG features of the sleep stages W, N1, N2, N-3, and R.

Neural Network		Prediction									
		N1	N2	N3	REM	Wake	N1	N2	N3	REM	Wake
**Actual**	**N1**	2197	2634	88	1948	2656	550	666	29	470	675
	**N2**	845	24,746	3078	1883	1038	196	6149	753	483	257
	**N3**	22	4250	12,647	42	174	7	1039	3066	13	30
	**REM**	699	2504	86	9331	537	176	624	21	2372	115
	**Wake**	980	796	68	318	13,284	243	212	23	78	3353

**Table 6:** Confusion matrix of the CHAID Model using training and testing datasets for the classification of EEG features of the sleep stages W, N1, N2, N-3, and R.

CHAID		Prediction									
		N1	N2	N3	REM	Wake	N1	N2	N3	REM	Wake
**Actual**	**N1**	2109	2946	270	1817	2381	541	741	54	451	603
	**N2**	1392	21,380	4679	3175	964	338	5305	1121	834	240
	**N3**	80	5835	10,913	147	160	16	1418	2659	31	31
	**REM**	1366	4547	422	6210	612	354	1152	103	1560	139
	**Wake**	1970	1697	199	479	11,101	535	407	66	124	2777

**Table 7:** Classification Performance parameters of the C5.0 Model using training and testing datasets for the classification of EEG features of the sleep stages W, N1, N2, N-3, and R.

C5.0	Training (Average Accuracy = 94%)					Testing (Average Accuracy = 87%)				
	Accuracy	Sensitivity	Specificity	Precision	Negative Predictive Value	Accuracy	Sensitivity	Specificity	Precision	Negative Predictive Value
**N1**	0.93	0.60	0.971	0.72	0.95	0.86	0.31	0.931	0.36	0.92
**N2**	0.89	0.87	0.903	0.84	0.93	0.78	0.73	0.817	0.69	0.84
**N3**	0.95	0.86	0.970	0.88	0.96	0.91	0.74	0.944	0.76	0.94
**R**	0.96	0.84	0.976	0.86	0.97	0.89	0.62	0.942	0.66	0.93
**W**	0.96	0.92	0.969	0.86	0.98	0.92	0.81	0.946	0.77	0.96

**Table 8:** Confusion matrix of the Neural Network Model using training and testing datasets for the classification of EEG features of the sleep stages W, N1, N2, N-3, and R.

Neural Network	Training (Average Accuracy = 89%)					Testing (Average Accuracy = 89%)				
	Accuracy	Sensitivity	Specificity	Precision	Negative Predictive Value	Accuracy	Sensitivity	Specificity	Precision	Negative Predictive Value
**N1**	0.89	0.23	0.97	0.46	0.91	0.89	0.23	0.97	0.47	0.91
**N2**	0.80	0.78	0.82	0.71	0.87	0.80	0.78	0.82	0.71	0.87
**N3**	0.91	0.74	0.95	0.79	0.94	0.91	0.74	0.95	0.79	0.94
**R**	0.91	0.71	0.94	0.69	0.95	0.91	0.72	0.94	0.69	0.95
**W**	0.92	0.86	0.94	0.75	0.97	0.92	0.86	0.94	0.76	0.97

**Table 9:** Confusion matrix of the CHAID Model using training and testing datasets for the classification of EEG features of the sleep stages W, N1, N2, N-3, and R.

CHAID	Training (Average Accuracy = 84%)					Testing (Average Accuracy = 84%)				
	Accuracy	Sensitivity	Specificity	Precision	Negative Predictive Value	Accuracy	Sensitivity	Specificity	Precision	Negative Predictive Value
**N1**	0.86	0.22	0.94	0.30	0.91	0.86	0.23	0.94	0.30	0.91
**N2**	0.71	0.68	0.73	0.59	0.80	0.71	0.68	0.73	0.59	0.80
**N3**	0.86	0.64	0.92	0.66	0.91	0.87	0.64	0.92	0.66	0.91
**R**	0.86	0.47	0.92	0.53	0.91	0.85	0.47	0.92	0.52	0.91
**W**	0.90	0.72	0.94	0.73	0.94	0.90	0.71	0.94	0.73	0.94

**Table 10:** Comparative analysis of the methods and outcomes of the proposed work with other sleep studies.

Study	Year	Study Subject	Dataset (Year)/Signal	Class	Algorithm	Accuracy %
Tzimourta et al. [40]	2018	100 subjects	ISRUC-Sleep dataset (2009–2013)/EEG	Five-class {W, N1, N2, N3, and REM}	Random Forest	75.29
Kalbkhani et al. [41]	2018	100 subjects	ISRUC-Sleep dataset (2009–2013)/EEG	Five-class {W, N1, N2, N3, and REM}	SVM	82.33
Tripathi et al. [42]	2020	25 subjects	Cyclic Alternating Pattern (CAP) (2001)/EEG	Six-class {W, S1, S2, S3, S4, and REM}	Hybrid Classifier	71.68
Widasari et al. [43]	2020	51 subjects	Cyclic Alternating Pattern (CAP) (2001)/EEG	Four-class {W, Light sleep (S1 + S2), Deep sleep (S3 + S4), and REM}	Ensemble of bagged tree (EBT)	86.26
Wang et al. [44]	2020	157 subjects	Sleep-EDF Expanded (Sleep-EDFX) (2000)/EEG and EOG	Five-class {W, N1, N2, N3, and REM}	Ensembles of EEGNet-BiLSTM	82
Sharma et al. [45]	2021	80 subjects	Cyclic Alternating Pattern (CAP) (2001)/EEG	Six-class {W, S1, S2, S3, S4, and REM}	Ensemble of Bagged Tree (EBT)	85.3
Proposed work	2022	157 subjects	HMC-Haaglanden Medisch Centrum (2021)/EEG	Five-class {W, N1, N2, N3, and REM}	C5.0, Neural Network, and CHAID	C5.0 (91%), Neural Network (92%), and CHAID (84%)
